# Tailoring a national emergency medical team training package for Pacific island countries and areas

**DOI:** 10.5365/wpsar.2023.14.6.1033

**Published:** 2023-12-15

**Authors:** Erin Elizabeth Noste, Anthony T Cook, Jan-Erik Larsen, Simon Cowie, Sean T Casey

**Affiliations:** a World Health Organization Regional Office for the Western Pacific, Manila, Philippines.; b Department of Emergency Medicine, University of California San Diego, San Diego, California, United States of America.; c School of Population Health, University of New South Wales, Sydney, New South Wales, Australia.

## BACKGROUND

Pacific island countries and areas (PICs) comprise thousands of populated islands spread across vast ocean territory, with some of the most challenging logistics in the world. Pacific populations are particularly vulnerable to disasters and the intersecting impacts of climate change. Recent sudden-onset disasters in the Pacific, such as Tropical Cyclone Yasa in Fiji in 2020 and the Hunga–Tonga Hunga–Ha’apai (HTHH) volcanic eruption and tsunami in the Kingdom of Tonga in 2022, have highlighted the need for national emergency medical teams (EMTs). These are clinical teams trained and equipped to deploy to and operate in remote island contexts, with the capability to respond independently to disasters within their borders and care for affected populations. (*1*, *2*) EMTs are groups of health professionals that treat patients impacted by emergencies and disasters, based on established global principles and standards of self-sufficiency, quality of care and coordination. (*3*)

Since 2017, Pacific ministries of health have been developing national EMTs to support health emergency responses within their borders and have trained team members to be ready for deployments. (*4*-*9*) Pacific EMTs are considered “Type 1” according to the standards in the World Health Organization (WHO) *Classification and Minimum Standards for Emergency Medical Teams* (2021), also known as the “Blue Book.” (*3*) Pacific EMTs vary in size and composition, with the smallest teams deploying only 6–10 team members at a time – adapting global EMT standards for the realities of small Pacific contexts.

With financial and technical support from partners, WHO’s Regional Office for the Western Pacific has facilitated EMT development and training across the Pacific since 2017. (*4*) This brief report describes how WHO has leveraged global and regional EMT training content and approaches, and adapted these for unique Pacific contexts.

### Training foundations

Curricula for EMT member training courses have traditionally been based on the minimum standards described in the Blue Book, with individual teams leading the design and delivery of training for their own team members. (*3*, *10*) The Pacific EMT training package applies Blue Book principles and standards, but training content has been specifically tailored to the unique small Pacific island contexts. Training materials developed by WHO and peer EMTs have been used as the foundation of the Pacific EMT member training package. Group activities, practical exercises and *talanoa* or storytelling discussions have been added, in addition to content on readiness for deployments to remote and difficult-to-reach islands to care for populations with limited on-site resources and referral options. (*11*) Hands-on activities and logistics sessions are based on the EMT cache (equipment) procured by WHO for Pacific EMTs. (*12*) Training content and approaches emphasize readiness for the deployment of light, mobile clinical response teams to disasters and outbreaks in PICs.

### Training content

The 5-day Pacific EMT member training programme comprises a mix of didactic and practical sessions, plus a 1–2-day simulation exercise (**Table 1**), with each session underpinned by learning objectives (**Table 2**). The training package is designed to provide information and develop skills in three EMT knowledge areas: (1) overview, administration and coordination; (2) clinical care; and (3) operational logistics and water, sanitation and hygiene or WASH. Training participants include clinicians, logisticians and administrators.

**Table 1:** Illustrative schedule for 5-day Pacific EMT member training course

Day 1	Day 2	Day 3	Day 4	Day 5
National EMT/WHO training official opening (45 minutes)	Morning review	Morning review	Morning review	Travel for SIMEX
	How is national EMT activated? (75 minutes)	EMTs in outbreak response (60 minutes)	Resilience on deployment (90 minutes)	SIMEX (4 hours minimum)
EMT initiative introduction (45 minutes)				
		EMT coordination (45 minutes)		
Coffee break (15–30 minutes)	Coffee break (15–30 minutes)	Coffee break (15–30 minutes)	Coffee break (15–30 minutes)	
Introduction to the Blue Book and national EMTs (75 minutes)	Clinical overview (90 minutes)	Logistics and WASH overview (90 minutes)	Triage and mass casualty management (60 minutes)	
Lunch (30–60 minutes)	Lunch (30–60 minutes)	Lunch (30–60 minutes)	Lunch (30–60 minutes)	Lunch (30–60 minutes)
Health standards, principles and ethics (90 minutes)	Conditions of service and standard operating procedures (90 minutes)	Site layout and camp planning (90 minutes)	Triage and mass casualty incident activity (90 minutes)	SIMEX debrief: self and peer feedback, review of key learning points
Coffee break (15–30 minutes)	Coffee break (15–30 minutes)	Coffee break (15–30 minutes)	Coffee break (15–30 minutes)	
Deployment cycle and national EMT structure (90 minutes)	Safety and security on deployment (45 minutes)	Feeding the team (60 minutes)	SIMEX briefing and preparation (60 minutes)	
	Team communication and radio procedures (45 minutes)			

EMT: emergency medical team; SIMEX: simulation exercise; WASH: water, sanitation and hygiene; WHO: World Health Organization.

**Table 2:** Pacific EMT member training course, learning objectives

Session title	Learning objectives
Introduction to the Global EMT Initiative	Explain the EMT core principles and minimum standards for self-sufficiency and quality care in emergency response
	Review the goals of the Global EMT Initiative and the importance of national, regional and international emergency response capacity
	Review team roles and responsibilities, including logistics, WASH and clinical roles
	Review the role of the EMT within the context of other health emergency responders
	Introduce experiences in the Pacific with national EMT deployments in response to emergencies
EMT life cycle	Introduce the concept of the EMT life cycle and the EMT’s role in a national response to a disaster
	Explain the activation and mobilization process of EMTs
	Discuss the planning, coordination and collaboration required for planning the EMT’s demobilization and exit
EMT coordination	Describe the different models for the EMT Coordination Cell (EMT-CC) and how the ministry of health or other government ministries play a role in the coordination of EMTs
	Introduce the process for requesting and coordinating international EMTs
	Discuss the importance of EMT reporting
	Discuss when national response capacity may need to be supplemented with international EMTs
Health standards, principles and ethics	Understand the EMT guiding principles and how they are applied to real-life examples
Clinical overview	Explain the importance of achieving minimum health standards of care during EMT operations
	Describe the existing standards of health care and health infrastructure both locally and regionally, including the relationship with EMT principles and standards
	Locate desired information within the national EMT standard operating procedures (SOPs) and the EMT Blue Book
	Describe how emergencies can increase medical risks for vulnerable people
	Outline the different deployment modalities a national EMT can be requested to perform (mobile clinic, surge support, community checks, evacuation centre staffing)
EMTs in outbreak response	Describe the roles of EMTs in outbreak response
	Use prior outbreak responses in the Pacific to highlight the important role EMTs play in outbreak response
	Describe the importance of reporting patient encounters and the use of the Minimum Data Set (MDS) in all EMT activities
	Demonstrate ability to use the MDS during an EMT deployment
Triage and MCIs	Recognize the approach in the management of multiple casualties within the EMT context
	Review the challenges of managing multiple casualties within the EMT
	Demonstrate how to effectively manage overwhelming numbers of patients encountered
	Describe what patients will need to be transferred from an EMT to national health facilities and other care centres
Resilience on deployment	Discuss previous experience with stress management
	Share what strategies for stress management worked well
	Using the deployment cycle, review predicted stressors and discuss individual and group mitigation strategies
EMT cache familiarization	Identify the core components of individual (non-clinical) field kits necessary for national EMT deployments
	Provide hands-on training on EMT deployment gear
**Session title**	Learning objectives
Logistics overview	Describe how team members and the logistics focal point must plan for team self-sufficiency while in the field
	Explain EMT field logistics and logistics operations in an emergency response
	Review team members’ roles in EMT logistics
WASH overview	Review how WASH will be approached in a mobile medical response
	Describe how water will be stored, tested and treated on EMT deployment
	Describe how medical and non-medical waste will be managed on a deployment
	List ways of vector control
Safety and security	Provide an overview of team member safety on a deployment
	Review safety measures when team is being transported by boat, road or air
	Describe safety rules for fire and fuel
	Understand the importance of water safety when deploying in the Pacific
Mobile medical care layout and camp planning	Provide an overview of how to choose an appropriate location to establish the EMT camp and how to set up gear
	Describe camp layout considerations
	Plan for mobile medical-care layout
Communication	Operate basic functions of emergency communications equipment that will be used in a deployment, including radios and satellite communication devices
	Understand the importance of effective radio communications and how to use correct radio terminology, including the phonetic alphabet
	State the purpose of a situation report (SITREP) and understand how to effectively write a SITREP for sharing with relevant authorities and coordination bodies
	Understand how to run daily briefing sessions with team members and utilize techniques to ensure their effectiveness

EMT: emergency medical team; MCI: mass casualty incident; WASH: water, sanitation and hygiene.

The overview, administration and coordination sessions introduce the EMT initiative with a specific emphasis on how the initiative was developed and how EMT principles and approaches have been applied in the Pacific to improve health emergency response.

Clinical sessions present the types of medical care provided in lightweight, mobile outpatient EMT clinics. Sessions incorporate team activities to discuss ethical issues during the care of patients in an emergency, as well as some of the clinical decisions to be made based on the logistical limitations of mobile medical care in remote island settings.

Logistics and WASH sessions provide hands-on activities using EMT equipment to prepare Pacific EMT members to operate with self-sufficiency. These sessions cover EMT camp planning and clinic set-up, day-to-day operations, clinic handover and responsible EMT exit. Specific concepts are underscored throughout the 5-day training, such as safety, reporting and radio/satellite communication protocols.

A simulation exercise is held on the final day of the training for a minimum of 4 hours, although this can be expanded to an overnight simulation exercise at a remote island location, where available. Given that most EMTs in the Pacific may encounter a disaster response with the need to utilize small boats, the simulation exercise often includes a practical session in which the teams load their EMT cache onto small boats and set up their camp and clinic in a remote location. Recent EMT training courses in Samoa and Vanuatu have used nearby uninhabited islands for these exercises.

### Training delivery

Following initial team member training in 2018–2019, in-person EMT training in the Pacific was paused due to the COVID-19 pandemic and extended border closures. In 2022, national EMT training courses recommenced and were held in Fiji, Kiribati, the Marshall Islands, Palau, Samoa and Vanuatu using the updated training package. Participants included physicians, nurses, public health experts, environmental health specialists, paramedics, firefighters, pharmacists, logisticians, and police and military personnel, depending on the national composition of each EMT. There were 20–30 participants at each training. In Fiji and Samoa, the participants stayed in *bures* or *fales* (thatched huts) in remote locations so that they were removed from their usual daily clinical tasks, which increased opportunities for team-building activities.

Feedback was obtained from the participants through anonymous online surveys after training sessions. Generally, the results of the survey were highly positive, particularly regarding interactive sessions on cache/equipment familiarization, group discussions on the logistics of response in a remote island setting, and full-scale simulation exercises, including the use of actors and real-world deployment modalities (for example, small seacraft).

## Discussion

Recent national EMT deployments in the Pacific, such as during the HTHH volcanic eruption and tsunami, highlighted the successful deployment of national EMTs and underscored the need for recurring national training tailored to Pacific contexts. In Tonga, the last EMT training before the HTHH deployment had been before the COVID-19 pandemic, and many of the deployed team members had not attended a formal training course. (*13*)

Tailoring training materials and approaches to specific country contexts is essential for knowledge and skills acquisition, and the application of those skills during deployments. In the Pacific, an emphasis on practical activities based on previous deployments in remote islands stimulates increased participant engagement, and training is viewed by participants as useful in preparing them for real-world deployments.
